# Identification and Validation of Reference Genes for Gene Expression Analysis in Different Development Stages of *Amylostereum areolatum*

**DOI:** 10.3389/fmicb.2021.827241

**Published:** 2022-01-12

**Authors:** Ningning Fu, Jiaxing Li, Ming Wang, Lili Ren, Shixiang Zong, Youqing Luo

**Affiliations:** Beijing Key Laboratory for Forest Pest Control, Beijing Forestry University, Beijing, China

**Keywords:** *Sirex noctilio*, *Amylostereum areolatum*, RT-qPCR, reference gene, growth and development

## Abstract

A strict relationship exists between the *Sirex noctilio* and the *Amylostereum areolatum*, which is carried and spread by its partner. The growth and development of this symbiotic fungus is key to complete the life history of the *Sirex* woodwasp. Real-time quantitative polymerase chain reaction (RT-qPCR) is used to measure gene expression in samples of *A. areolatum* at different growth stages and explore the key genes and pathways involved in the growth and development of this symbiotic fungus. To obtain accurate RT-qPCR data, target genes need to be normalized by reference genes that are stably expressed under specific experimental conditions. In our study, the stability of 10 candidate reference genes in symbiotic fungal samples at different growth and development stages was evaluated using geNorm, NormFinder, BestKeeper, delta Ct methods, and RefFinder. Meanwhile, *laccase1* was used to validate the stability of the selected reference gene. Under the experimental conditions of this study, *p450*, *CYP*, and γ*-TUB* were identified as suitable reference genes. This work is the first to systematically evaluate the reference genes for RT-qPCR results normalization during the growth of this symbiotic fungus, which lays a foundation for further gene expression experiments and understanding the symbiotic relationship and mechanism between *S. noctilio* and *A. areolatum*.

## Introduction

*Sirex noctilio* Fabricius (Hymenoptera; Symphyta; and Siricidae) is an important forest woodborer, which mainly damages *Pinus* species (Slippers et al., 2012) worldwide. In China, it was first reported in Daqing, Heilongjiang Province in July 2013, seriously harming *Pinus sylvestris* var. *mongolica* plantations in many regions of the country (Slippers et al., 2012; Li et al., 2015; Sun et al., 2016; Wang et al., 2018). *Amylostereum areolatum* (Fr.) Boidin (Basidiomycotina: Corticiaceae) is a symbiotic fungus of the woodwasp, and both exist in a strictly mutualistic relationship (Biedermann and Vega, 2020; Wang M. et al., 2020; Li et al., 2021). *S. noctilio* carries the oidia and mycelia of *A. areolatum* through the specialized mycangium, which disperses and introduces the fungus into a new host during oviposition (Slippers et al., 2012; Li et al., 2015). Concurrently, *A. areolatum* digests and degrades large molecular carbohydrates to small ones in the xylem of pine wood by secreting extracellular enzymes, which provides necessary nutritional for the incubation of woodwasp eggs and larval growth and development (Talbot, 1977; Madden and Coutts, 1979; Hajek et al., 2013; Wang et al., 2019). Before the third or fourth instar stages, the woodwasp larvae obtain nutrition by directly feeding on the mycelia of the symbiotic fungus, and then on the xylem infected by *A. areolatum* (Madden and Coutts, 1979; Thompson et al., 2014). In addition, Wang L. et al. (2020) suggested that when the incidental fungi in the host tree inhibited the growth of the symbiotic fungus, the mortality of woodwasp larvae increased significantly. Therefore, we believe that the growth and development of *S. noctilio* larvae are directly affected by the growth of *A. areolatum*.

Real-time quantitative polymerase chain reaction (RT-qPCR) is a widely used method for quantitative analysis of gene expression due to its accuracy in quantification, repeatability and high sensitivity (Bustin et al., 2005; Engel et al., 2007; Gao et al., 2020). Nevertheless, the accuracy of RT-qPCR results depends on many factors, including the quality and quantity of initial RNA, primer specificity, amplification efficiency, and transcript normalization, to name a few (Nolan et al., 2006; Kozera and Rapacz, 2013). Among these, normalization is a key factor affecting the RT-qPCR results (Udvardi et al., 2008; Yang et al., 2021). We usually need to introduce reference genes to correct and normalize the expression of target genes. Due to the spatio-temporal specificity of gene expression, a suitable reference gene should be stably and effectively expressed under specific experimental conditions (Lv et al., 2020; Wang G. et al., 2020). Therefore, screening appropriate reference genes for specific experimental materials or experimental conditions is important for RT-qPCR analysis. In fungi, *18S* (18S ribosomal RNA), *28S* (28S ribosomal RNA), *GAPDH* (glyceraldehyde-3-phosphate hydrogenase), *ACT* (actin), *EF1-*α (elongation factor1-α), *RPB2* (RNA polymerase subunit 2), *CYP* (cyclophilin), β-*TUB* (β-tubulin), *CYT b* (cytochrome b), and other genes have been often used as reference genes (Lian et al., 2014; Zhang et al., 2016; Jia et al., 2019; Singh et al., 2019; Song et al., 2019; Yang et al., 2019). While the selection of reference genes has been reported in fungi such as *Ganoderma lingzhi* (Xu et al., 2014), *Pleurotus eryngii* (Qin and Wang, 2015), *Auricularia cornea* (Jia et al., 2019), *Auricularia heimuer* (Zhang et al., 2020), *Pleurotus ostreatus* (Fernández-Fueyo et al., 2014), and *Lentinula edodes* (Xiang et al., 2018), there are no reports on the reference genes in *A. areolatum*. Therefore, we need to find appropriate reference genes for gene expression analysis of this symbiotic fungus.

To study the key genes involved in the growth and development of *A. areolatum*, we selected 10 candidate genes with relatively stable expression from its genome and transcriptome data. We then analyzed the expression levels of these genes in the samples of *A. areolatum* at different growth and development stages. In order to select suitable reference genes, some algorithms and software were used to comprehensively evaluate the expression stability of these genes. The findings of this study will contribute to the study on the mechanism of growth and development of *A. areolatum*, and provide a reference basis for further exploring its symbiotic relationship with *S. noctilio*.

## Materials and Methods

### Strains and Sample Collection

*Amylostereum areolatum* was isolated from four female woodwasps collected from *P. sylvestris* var. *mongolica* from the Jun De Forest Farm, Heilongjiang Province, China. After identification, the fungus was preserved at the Beijing Key Laboratory for the Control of Forest Pest, Beijing Forestry University, Beijing, China. *A. areolatum* samples were first cultured at 25°C on potato dextrose agar (PDA) plates for 1 week, and then five mycelial plugs (6 mm diameter) were removed from the periphery of the mycelium and inoculated in 100 mL potato dextrose broth (PDB) medium. The mycelia were allowed to grow for 7 days at 25°C, and then collected at 12 h, 1, 3, 5, and 7 days. All samples were immediately frozen in liquid nitrogen and stored at −80°C for RNA isolation.

### Total RNA Extraction and cDNA Synthesis

Total RNA was extracted from samples of the fungus at different growth stages using EZgene TM Fungal RNA Kit (Biomiga, United States). The quality and quantity of total RNA were assessed by NanoDrop 2000 and electrophoresis on 1.0% agarose gels. Before reverse transcription, total RNA samples were pre-treated by gDNA Eraser (Takara, Dalian, China) to further eliminate DNA in samples. Then, we used the PrimeScript TM RT reagent Kit (Takara, Dalian, China) to synthesize the first strand cDNA according to the manufacturer’s protocol. One microgram of total RNA was used to synthesize cDNA in a 20 μL reaction system, and then diluted 10-fold for RT-qPCR.

### Candidate Reference Genes Selection and Primer Design

A total of ten stably expressing candidate reference genes of *A. areolatum* in its different growth and development stages were selected based on the transcriptome (BioProject: PRJNA733162; BioSample: SAMN19369336–SAMN19369343) and genome (GenBank: SAXG00000000; BioProject: PRJNA513942) data (Fu et al., 2020, 2021). These genes were α*-TUB*, β*-TUB*, γ*-TUB*, *GAPDH*, *CYP*, *CPR* (NADPH-dependent cytochrome P450 oxidoreductase), *HH3* (Histone H3), *CESP* (coatomer epsilon subunit-domain-containing protein), *MDP* (metallo-dependent phosphatase), and *P450* (cytochrome P450). The CDS sequences of these genes were obtained from the *A. areolatum* genome data (GenBank: OL660755–OL660764), and the primers were designed using the web software IDT^1^ and Primer 3.0^2^. The primers used for amplification are listed in Table 1.

**TABLE 1:** Primer sequences and amplification characteristics of the candidate reference genes.

Symbol	Gene name	Primer (5′-3′)	Size (bp)	Efficiency%	*R*^2^ value
α*-TUB*	α-tubulin	F: CGTGTTTCGAGAGCGGTAAT	114	90.4	0.992
		R: GGATCGTGCGCTTAGTCTTAAT			
β*-TUB*	β-tubulin	F: CATTGACAACGAGGCTCTCTAC	99	97.0	0.992
		R: GACATGACGATGGAAACGAGAT			
γ*-TUB*	γ-tubulin	F: TGTGTCGGCATGATTGAGAG	102	96.5	0.998
		R: TGCGTGTGATTGAGCATTTAAC			
*GAPDH*	Glyceraldehyde-3-phosphate hydrogenase	F: GACTCCAAGTACACCGTCATC	95	98.4	0.994
		R: TCGACGATGCCGAACTTATC			
*CYP*	Cyclophilin	F: CTCGATGACGGGTTTGGATATAA	75	105.7	0.987
		R: GTCACCACCCTGGATCATAAA			
*CPR*	NADPH-dependent cytochrome P450 oxidoreductase	F: CGTCGATCCCTTGAGAAGAAC	114	103.1	0.995
		R: CGGCCACTCATCACTGTAAA			
*HH3*	Histone H3	F: ACAAGACTTCAAGACGGATCTC	98	96.9	0.994
		R: TGGTGTCCTCGAACAATGAG			
*CESP*	Coatomer epsilon subunit-domain-containing protein	F: ACAAGACGATGCGGAAACA	107	100.7	0.993
		R: CGGCATATTTGGAAACGAACC			
*MDP*	Metallo-dependent phosphatase	F: AGCACAATGACGGGATGTT	101	109.7	0.994
		R: CCCATTTCCGACGACTTTGA			
*P450*	Cytochrome P450	F: CACCTTTGCAGTCTACCTACTT	118	107.1	0.996
		R: CAGCTCCTTCAGATCGTCTATG			

### Real-Time Quantitative Polymerase Chain Reaction Amplification

Real-time quantitative polymerase chain reaction amplification was performed using a Bio-Rad CFX Connect real-time PCR instrument (BIO-RAD, United States) with TB Green ^®^ Premix Ex Taq™ II (Takara, Japan). The amplification was performed in a 25 μL reaction volume according to the manufacturer’s instructions. Each reaction contained 12.5 μL of TB Green Premix Ex Taq II (2×), 1 μL of forward and reverse primers (10 μM), 2 μL of cDNA template (25 ng/μL) and 8.5 μL of RNAse free water. The amplification was conducted as follows: 95°C for 30 s; 40 cycles of 95°C for 5 s and 60°C for 30 s. A single melting curve from 65 to 95°C (increments of 0.5°C every 5 s) was performed to verify the primer specificity. Four biological (each with a different isolate) and three technical replicates per isolate were performed independently. The relative standard curve for each candidate gene was analyzed using a 10-fold dilution series (1, 1/10, 1/100, 1/1,000, and 1/10,000) of cDNA as template and the amplification efficiency was calculated using the slope of the linear regression equation: E% = [10(−1/slope)−1] × 100%.

### Stability Analysis of Candidate Reference Genes

The cycle threshold (*Ct*) values of amplification of genes in samples from different growth and development stages of *A. areolatum* were collated and summarized using Microsoft Excel. The stability of the ten candidate reference genes was analyzed by geNorm (v 3.5) (Vandesompele et al., 2002), NormFinder (v 0.953) (Andersen et al., 2004), BestKeeper (Pfaffl et al., 2004), RefFinder^3^ (Xie et al., 2012), and delta Ct method (Silver et al., 2006). For geNorm, the expression stability value (M) of the candidate reference gene was calculated according to the pairwise variation (V) between two candidate genes. Candidate reference genes with lower *M* values were more stable. Additionally, the optimal numbers of reference genes were determined by calculating the pairwise variation V_n_/V_n + 1_. When this value was less than 0.15, *n* reference genes were needed to ensure the accurate normalization of RT-qPCR data (Vandesompele et al., 2002). To estimate intra- and inter- group variations between samples, NormFinder was used based on the linear mixed effect model. Stable candidate reference genes had lower *S* values (Andersen et al., 2004). The stability of candidate reference genes was analyzed by GeNorm and NormFinder using the 2^–ΔCt^ values, of which ΔCt = average Ct of the sample—minimum Ct of all RT-qPCR data (Zhao et al., 2020). The standard deviation (SD) and stability value (SV) of candidate reference genes were directly calculated by BestKeeper using the original *Ct* value to evaluate the stability of these genes (Pfaffl et al., 2004). The genes with lower SD and SV values had a more stable expression. Similarly, the delta Ct method was used to calculate the mean SD of the paired genes in each sample to verify the stability of their expression. Finally, RefFinder integrated these four methods to rank the candidate reference genes and evaluate the comprehensive stability of these genes (Xie et al., 2012).

### Validation of Selected Reference Genes

Based on the transcriptome data, *laccase1* was used to verify the stability of the selected reference genes. The primers used for amplification of *laccase1* were F (5′-3′: GTAGGAGCA CGTCCATTCATT) and R (5′-3′: TTGTAGAGGAACGTC GCATTT). The expression levels of *laccase1* in *A. areolatum* samples were detected by RT-qPCR using the same procedure as mentioned above and the most stable and unstable reference genes were used to normalize the expression levels of *laccase1* in different samples. The relative expression of genes was calculated using the 2^–ΔΔCt^ method, followed by taking its square root. The one-way analysis of variance (ANOVA) with the *post hoc* Tukey’s-HSD test was used to evaluate the effect on the expression of target genes by different reference genes (Florez et al., 2020).

## Results

### Primer Performance During Analysis of Candidate Reference Genes

The specificity of 10 candidate reference gene primers was determined by resolving the amplified products using 2% agarose gel electrophoresis, and a specific band of about 100 bp was obtained for each gene (Supplementary Figure 1). Consistently, the RT-qPCR melting curves showed that each reference gene produced a single peak, with no primer dimer or non-specific amplification (Supplementary Figure 2). In addition, the efficiency of RT-qPCR amplification of 10 candidate reference genes was between 90.4 and 109.7%, and the linear correlation coefficient (*R*^2^) was greater than 0.99 (except for *CYP*, which was 0.987) (Table 1). This suggested that all the primers for amplification of these genes were specific and efficient, and could meet the requirements of reference genes analysis.

### Expression Analysis of the Reference Genes

**FIGURE 1:**
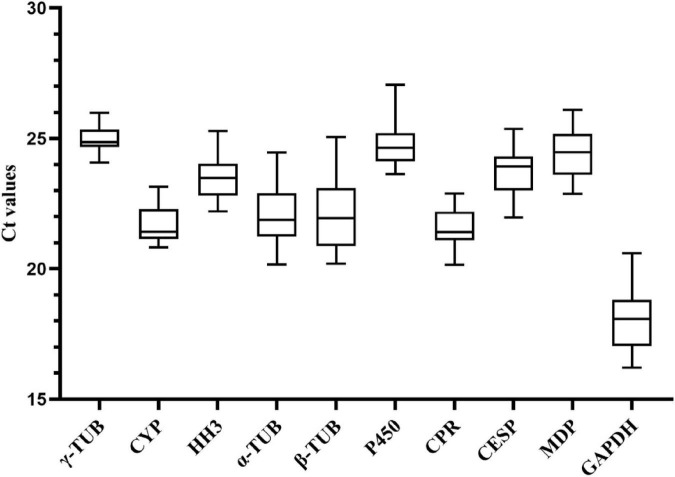
Cycle threshold (*Ct*) values of 10 candidate reference genes across all samples at different development stages. The box indicates the 25th and 75th percentiles and the line in the box represents the median value.

We analyzed the expression levels of 10 candidate reference genes in samples of different growth and development stages of *A. areolatum* by RT-qPCR. Average *Ct* values of these genes were in the range of 18.04–25.00 (Figure 1). Among these, the gene with the highest expression was *GAPDH* (average Ct = 18.04), and that with the lowest expression was γ*-TUB* (average Ct = 25.00). According to *SD* values, the genes γ*-TUB* (average Ct ± SD = 25.00 ± 0.48), *CPR* (average Ct ± SD = 21.54 ± 0.74), and *CYP* (average Ct ± SD = 21.75 ± 0.79) showed low variation in expression in the analyzed samples, while the genes β*-TUB* (average Ct ± SD = 22.05 ± 1.41), *GAPDH* (average Ct ± SD = 18.04 ± 1.23), and α*-TUB* (average Ct ± SD = 22.10 ± 1.13) showed the highest variation.

### Stability Analysis of Candidate Reference Genes

**FIGURE 2:**
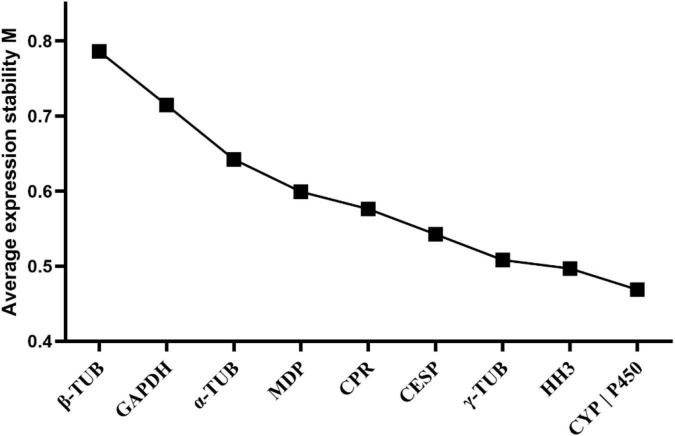
Average expression stability and ranking of the candidate reference genes calculated by geNorm. Candidate reference genes with lower *M* values were more stable. The least stable genes are listed on the left and the most stable genes are listed on the right.

In geNorm analysis, reference genes with *M* values less than 1.5 were generally considered to be stable. In our study, the *M* values of candidate reference genes evaluated by geNorm were all less than 1.5, indicating relatively stable expression of these genes. Among these, *CYP* and *P450* exhibited the most stable expression in those analyzed samples, while β*-TUB* was the most unstable gene (Figure 2 and Table 2).

**TABLE 2:** Expression stability ranking of the 10 candidate reference genes based on five algorithms.

Reference genes	geNorm		NormFinder		BestKeeper			delta Ct		RefFinder	
	M	Rank	SV	Rank	SD	CV	Rank	Avg. Ct	Rank	GM	Rank
*P450*	0.47	1	0.26	2	0.64	2.57	3	0.66	1	1.57	1
*CYP*	0.47	1	0.24	1	0.67	3.1	5	0.67	2	1.78	2
γ*-TUB*	0.51	4	0.34	5	0.38	1.52	1	0.73	4	2.99	3
*HH3*	0.50	3	0.29	3	0.66	2.82	4	0.70	3	3.22	4
*CPR*	0.58	6	0.35	6	0.58	2.68	2	0.75	6	4.56	5
α*-TUB*	0.64	8	0.32	4	0.91	4.13	8	0.74	5	5.98	6
*CESP*	0.54	5	0.45	8	0.72	3.03	6	0.82	8	6.62	7
*MDP*	0.60	7	0.36	7	0.82	3.37	7	0.77	7	7.00	8
*GAPDH*	0.72	9	0.54	9	0.94	5.22	9	0.94	9	9.00	9
β*-TUB*	0.79	10	0.66	10	1.15	5.23	10	1.07	10	10.00	10

*M, expression stability value; SV, stability value; SD, standard deviation; CV, coefficient of variation; Avg. Ct, average Ct; GM, geometric mean.*

**FIGURE 3:**
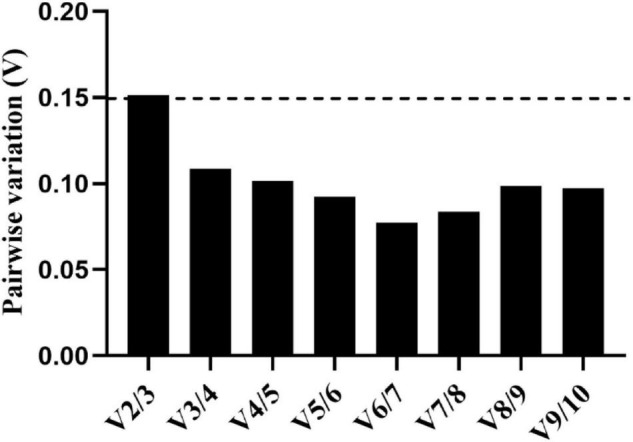
Pairwise variation (V) of 10 reference genes calculated by geNorm. The threshold value for assessing the optimal number of reference genes for Real-time quantitative polymerase chain reaction (RT-qPCR) normalization is 0.15.

The optimal number of reference genes was estimated using the pairwise variation (V_n_/V_n + 1_) calculated by geNorm. In our study, the value of V2/V3 was 0.151, which was greater than the cut-off value of 0.15, while other pairwise variation values were all less than 0.15 (Figure 3). Therefore, under the experimental conditions in this study, three reference genes were needed to normalize the target genes.

For NormFinder, it evaluates the stability of candidate genes based on an ANOVA-based algorithm. Under our experimental conditions, the order of stability of reference genes evaluated by NormFinder software was *CYP* > *P450* > *HH3* > α*-TUB* > γ*-TUB* > *CPR* > *MDP* > *CESP* > *GAPDH* > β*-TUB* (Table 2).

The stability of the candidate reference genes was evaluated using the BestKeeper by directly calculating the SD and CV of *Ct* values of these genes. According to the evaluation results, γ*-TUB*, *CPR*, and *P450* were the most stable genes in the *A. areolatum* samples at different growth and development stages, while α*-TUB*, *GAPDH*, and β*-TUB* were poorly stable. In particular, β*-TUB* had an *SD* value greater than 1 and was thus considered to be an unstable reference gene (Table 2).

We also used the delta Ct method to evaluate the stability of the candidate reference genes. The average SD of these genes ranged from 0.66 to 1.07 (Table 2). As a result, *P450* was the most stable reference gene, while β*-TUB* was the most unstable gene in the symbiotic fungus samples at different growth and development stages.

The stability rankings of the 10 candidate reference genes evaluated by geNorm, NormFinder, BestKeeper, and delta Ct were not completely consistent (Figure 2 and Table 2). For a more accurate analysis, we used the comprehensive analysis software RefFinder to calculate the geometric mean of the results for each gene obtained by geNorm, NormFinder, BestKeeper, and delta Ct to provide a comprehensive ranking of the reference genes. The candidate genes evaluated by RefFinder were ranked from the most to the least stable as: *P450* > *CYP* > γ*-TUB* > *HH3* > *CPR* > α*-TUB* > *CESP* > *MDP* > *GAPDH* > β*-TUB*. According to the optimal number calculated by geNorm and the reference gene ranking obtained by RefFinder, the optimal reference gene combination under our experimental conditions was *P450* + *CYP* + γ*-TUB*.

### Validation of the Selected Reference Genes

**FIGURE 4:**
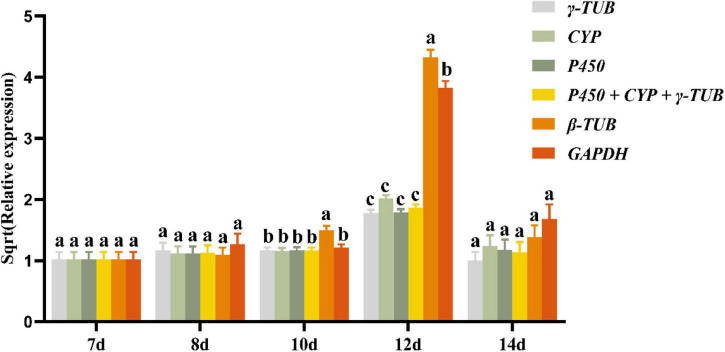
Relative expression levels of *laccase1* normalized by *P450*, *CYP*, γ*-TUB*, *GAPDH*, and β*-TUB*. Different letters indicate the significant differences of *laccase1* expression levels [analysis of variance (ANOVA), HSD, *p* < 0.05]. Sqrt (Relative expression) represents the square root of the relative expression value.

To verify the reliability of the selected reference genes, the expression levels of *laccase1* in the above-analyzed samples were normalized using the three most stable candidate reference genes (*P450*, *CYP*, and γ*-TUB*), the combination of these stable genes (*P450* + *CYP* + γ*-TUB*), and the two most unstable reference genes (*GAPDH* and β*-TUB*). We found that when *P450*, *CYP*, and γ*-TUB* and their combinations were used as reference genes, the expression levels of *laccase1* in the same sample were nearly consistent. When β*-TUB* was used as the reference gene, the expression levels of *laccase1* in the *A. areolatum* samples grown for 10 and 12 days were significantly different from the normalized results of other reference genes (Figure 4). Similarly, the expression levels of *laccase1* normalized by *GAPDH* also showed significant differences in 12-day old samples. The expression of *laccase1* in samples at different growth and development stages normalized by *P450*, *CYP*, and γ*-TUB* as well as their combination was consistent with the results of previous transcriptome studies (Fu et al., 2021). These findings indicated that these three genes individually or in combination were reliable as reference genes under our experimental conditions.

## Discussion

There is a strict mutualism between *S. noctilio* and *A. areolatum* (Slippers et al., 2012; Biedermann and Vega, 2020). The growth and development of this symbiotic fungus in host trees play an important role in the life history of the woodwasp (Li et al., 2015). A previous study showed that when the growth of the symbiotic fungus was restricted, the mortality rate of woodwasp larvae increased significantly (Wang L. et al., 2020). Thus, the growth of the *A. areolatum* is crucial for the growth and development of the *S. noctilio* larvae. At present, the genome (Fu et al., 2020) and transcriptome (Fu et al., 2021) sequencing of the *A. areolatum* has been completed by our laboratory; the obtained data provide a possibility for the analysis of key genes in the growth and development of this symbiotic fungus. These transcriptome data must be verified by RT-qPCR and to obtain effective amplification results, appropriate reference genes are required to normalize the target genes, as well as to reduce the experimental or inter-sample errors. Studies have found that there were no consistently expressed reference genes in different strains or under different experimental conditions. For example, Wang G. et al. (2020) showed that in *Tolypocladium guangdongense*, the vacuolar protein sorting gene *VPS* was the most stable gene under different developmental stages and temperature stress, while *H4* was the most stable gene under different carbon sources. In addition, *18S rRNA* and *28S rRNA* were stably expressed in different strains of *A. heimuer* (A14, A137, and A12) and at different growth stages (mycelium, protoplasm, and fruiting stages) (Zhang et al., 2020). Similarly, the expression of *18S rRNA* was also stable in *L. edodes* (Xiang et al., 2018)and *Wolfiporia cocos* (Zhao, 2016). However, the stability of *18S rRNA* and *28S rRNA* expression was relatively poor in *A. cornea* (Jia et al., 2019) and *Ganoderma lucidum* (Xu et al., 2014). Thus, suitable reference genes must be screened based on different species and different experimental conditions.

To avoid the limitations of using a single software, we used multiple reference gene stability assessment software (geNorm, NormFinder, BestKeeper, and RefFinder) to analyze the expression stability of candidate reference genes. Due to the use of different algorithms, the results of reference gene stability did not agree with each other. Similar differences have been reported in earlier studies (Jia et al., 2019; Lv et al., 2020; Yang et al., 2021). Thus, RefFinder was used to ensure the accuracy of the screening results by integrating these results of the above-mentioned four algorithms, and comprehensively evaluating the reference genes. A few earlier studies showed that under certain experimental conditions, the normalization of target gene expression using a single reference gene sometimes may not ensure the accuracy of experimental results (Jia et al., 2019; Wu et al., 2021). Therefore, two or more reference genes should be introduced to meet the experimental requirements. In our study, according to the optimal number calculated by geNorm and the reference gene ranking obtained by RefFinder, *P450*, *CYP*, and γ*-TUB* were identified as stable reference genes. One of these reference genes, γ*-TUB*, with an important role in the cytoskeleton, was also one of the most stable reference genes in different strains of *Cryptolestes ferrugineus* (Tang et al., 2017). In our research, the commonly used reference genes *CYP* and *GAPDH* were the most stable and unstable expressed genes, respectively. Likewise, *CYP* was the most unstable reference gene in *Volvariella volvacea* (Tao et al., 2016), while *GAPDH* was the most stable reference gene in *A. heimuer* (Zhang et al., 2020). These results again demonstrated that reference genes differed from species to species.

Laccase is involved in spore formation, pathogenesis, pigmentation, fruiting body formation and melanin formation in fungi (Camarero et al., 2005). It showed different expression levels at different developmental stages of fungi and was often used for reference gene verification (Jia et al., 2019). In our study, the expression profile of *laccase1* of *A. areolatum* at different growth stages was used to verify the stability of the putative reference gene. We found that in some samples, the expression of *laccase1* normalized by *GAPDH* and β*-TUB* was significantly different from the normalized results obtained using other reference genes. Thus, the normalization results based on *GAPDH* and β*-TUB* did not accurately reflect the expression level of *laccase1*. The expression levels of *laccase1* normalized by reference genes *P450*, *CYP*, and γ*-TUB*, as well as their combination were consistent in samples even at different growth stages. Therefore, these three genes alone, or in combination, could be used for RT-qPCR analysis of gene expression in *A. areolatum* under our experimental conditions.

This is the first study to evaluate reference genes for analysis of gene expression in *A. areolatum*. We evaluated the stability of 10 candidate reference genes in samples from this symbiotic fungus at different growth stages using geNorm, NormFinder, BestKeeper, delta Ct methods, and RefFinder. Overall, *P450*, *CYP*, and γ*-TUB* and their combination were the most stable reference genes in different samples, although the rankings of their stability varied slightly when analyzed by the four programs used here. Our results not only provided stable reference genes for quantitative analysis of gene expression of the symbiotic fungus, but also laid a foundation for the study of its mutualistic relationship with *S. noctilio*.

## Data Availability Statement

The datasets presented in this study can be found in online repositories. The names of the repository/repositories and accession number(s) can be found below: https://www.ncbi.nlm.nih.gov/genbank/, OL660755; https://www.ncbi.nlm.nih.gov/genbank/, OL660756; https://www.ncbi.nlm.nih.gov/genbank/, OL660757; https://www.ncbi.nlm.nih.gov/genbank/, OL660758; https://www.ncbi.nlm.nih.gov/genbank/, OL660759; https://www.ncbi.nlm.nih.gov/genbank/, OL660760; https://www.ncbi.nlm.nih.gov/genbank/, OL660761; https://www.ncbi.nlm.nih.gov/genbank/, OL660762; https://www.ncbi.nlm.nih.gov/genbank/, OL660763; and https://www.ncbi.nlm.nih.gov/genbank/, OL660764.

## Author Contributions

NF carried out the majority of the bioinformatics studies and participated in performing the experiments. MW was involved in experimental data analysis. NF and JL wrote the manuscript. LR, YL, and SZ participated in the design of the study and helped to draft the manuscript. All authors have read and agreed to the published version of the manuscript.

## Conflict of Interest

The authors declare that the research was conducted in the absence of any commercial or financial relationships that could be construed as a potential conflict of interest.

## Publisher’s Note

All claims expressed in this article are solely those of the authors and do not necessarily represent those of their affiliated organizations, or those of the publisher, the editors and the reviewers. Any product that may be evaluated in this article, or claim that may be made by its manufacturer, is not guaranteed or endorsed by the publisher.

## Footnotes

1
https://sg.idtdna.com/pages

2
https://bioinfo.ut.ee/primer3-0.4.0/

3
http://blooge.cn/RefFinder/

## References

[B1] Andersen C. L. Jensen J. L. Ørntoft T. F. (2004). Normalization of real-time quantitative reverse transcription-PCR data: a model-based variance estimation approach to identify genes suited for normalization, applied to bladder and colon cancer data sets. *Cancer Res.* 64 5245–5250. 10.1158/0008-5472 15289330

[B2] Biedermann P. H. Vega F. E. (2020). Ecology and evolution of insect–fungus mutualisms. *Annu. Rev. Entomol.* 65 431–455. 10.1146/annurev-ento-011019-024910 31610133

[B3] Bustin S. Benes V. Nolan T. Pfaffl M. (2005). Quantitative real-time RT-PCR–a perspective. *J. Mol. Endocrinol.* 34 597–601. 10.1677/jme.1.01755 15956331

[B4] Camarero S. Ibarra D. Martínez M. J. Martínez ÁT. (2005). Lignin-derived compounds as efficient laccase mediators for decolorization of different types of recalcitrant dyes. *Appl. Environ. Microbiol.* 71 1775–1784. 10.1128/AEM.71.4.1775-1784.2005 15812000 PMC1082544

[B5] Engel H. Kueppers C. Koenig M. Loeffert D. (2007). Successful gene expression analysis by multiplex, real-time, one-step RT-PCR, irrespective of the targets amplified. *Biotechniques* 43 230–231. 10.2144/000112560 17824392

[B6] Fernández-Fueyo E. Castanera R. Ruiz-Dueñas F. J. López-Lucendo M. F. Ramírez L. Pisabarro A. G. (2014). Ligninolytic peroxidase gene expression by *Pleurotus ostreatus*: differential regulation in lignocellulose medium and effect of temperature and pH. *Fungal Genet. Biol.* 72 150–161. 10.1016/j.fgb.2014.02.003 24560615

[B7] Florez L. M. Scheper R. W. Fisher B. M. Sutherland P. W. Templeton M. D. Bowen J. K. (2020). Reference genes for gene expression analysis in the fungal pathogen *Neonectria ditissima* and their use demonstrating expression up-regulation of candidate virulence genes. *PLoS One* 15:e0238157. 10.1371/journal.pone.0238157 33186359 PMC7665675

[B8] Fu N. Wang M. Gao C. Ren L. Luo Y. (2021). Transcriptomics analysis of *Amylostereum areolatum* at different development stages. *Mycosystema* 40 2771–2784. 10.13346/j.mycosystema.210208

[B9] Fu N. Wang M. Wang L. Luo Y. Ren L. (2020). Genome sequencing and analysis of the fungal symbiont of *Sirex noctilio*, *Amylostereum areolatum*: revealing the biology of fungus-insect mutualism. *msphere* 5 e00301–e00320. 10.1128/mSphere.00301-20 32404513 PMC7227769

[B10] Gao P. Wang J. Wen J. (2020). Selection of reference genes for tissue/organ samples of adults of *Eucryptorrhynchus scrobiculatus*. *PLoS One* 15:e0228308. 10.1371/journal.pone.0228308 32012184 PMC6996836

[B11] Hajek A. E. Nielsen C. Kepler R. M. Long S. J. Castrillo L. (2013). Fidelity among *Sirex woodwasps* and their fungal symbionts. *Microbial Ecol.* 65 753–762. 10.1007/s00248-013-0218-z 23532503 PMC3622004

[B12] Jia D. Wang B. Li X. Tan W. Gan B. Peng W. (2019). Validation of reference genes for quantitative gene expression analysis in *Auricularia cornea*. *J. Microbiol. Methods* 163:105658. 10.1016/j.mimet.2019.105658 31251967

[B13] Kozera B. Rapacz M. (2013). Reference genes in real-time PCR. *J. Appl. Genet.* 54 391–406. 24078518 10.1007/s13353-013-0173-x PMC3825189

[B14] Li D. Shi J. Lu M. Ren L. Zhen C. Luo Y. (2015). Detection and identification of the invasive *Sirex noctilio* (Hymenoptera: Siricidae) fungal symbiont, *Amylostereum areolatum* (Russulales: Amylostereacea), in China and the stimulating effect of insect venom on laccase production by *A. areolatum* YQL03. *J. Econ. Entomol.* 108 1136–1147. 10.1093/jee/tov072 26470239

[B15] Li H. Young S. E. Poulsen M. Currie C. R. J. (2021). Symbiont-mediated digestion of plant biomass in fungus-farming insects. *Annu. Rev. Entomol.* 66 297–316. 10.1146/annurev-ento-040920-061140 32926791

[B16] Lian T. Yang T. Liu G. Sun J. Dong C. (2014). Reliable reference gene selection for *Cordyceps militaris* gene expression studies under different developmental stages and media. *FEMS Microbiol. Lett.* 356 97–104. 10.1111/1574-6968.12492 24953133

[B17] Lv Y. Li Y. Liu X. Xu K. (2020). Identification of Ginger (Zingiber officinale Roscoe) reference genes for gene expression analysis. *Front. Genet.* 11:586098. 10.3389/fgene.2020.586098 33240331 PMC7670040

[B18] Madden J. Coutts M. (1979). “The role of fungi in the biology and ecology of woodwasps (Hymenoptera: Siricidae),” in *Insect-Fungus Symbiosis: Nutrition, Mutualism and Commensalism*, ed. Batra L. R. (Montclair, NJ: Halsted Press), 165–174.

[B19] Nolan T. Hands R. E. Bustin S. A. (2006). Quantification of mRNA using real-time RT-PCR. *Nat. Protoc.* 1 1559–1582. 10.1038/nprot.2006.236 17406449

[B20] Pfaffl M. W. Tichopad A. Prgomet C. Neuvians T. P. (2004). Determination of stable housekeeping genes, differentially regulated target genes and sample integrity: bestkeeper–excel-based tool using pair-wise correlations. *Biotechnol. Lett.* 26 509–515. 10.1023/b:bile.0000019559.84305.47 15127793

[B21] Qin X. Wang J. (2015). Selection of reference gene for quantitative real-time PCR analysis of lignification related genes in postharvest *Pleurotus eryngii*. *J. Northw. AF Univ.* 43 219–227. 10.13207/j.cnki.jnwafu.2015.07.024

[B22] Silver N. Best S. Jiang J. Thein S. L. (2006). Selection of housekeeping genes for gene expression studies in human reticulocytes using real-time PCR. *BMC Mol. Biol.* 7:33. 10.1186/1471-2199-7-33 17026756 PMC1609175

[B23] Singh S. Gupta M. Pandher S. Kaur G. Goel N. Rathore P. (2019). RNA sequencing, selection of reference genes and demonstration of feeding RNAi in *Thrips tabaci* (Lind.)(Thysanoptera: Thripidae). *BMC Mol. Biol.* 20:6. 10.1186/s12867-019-0123-1 30777032 PMC6380046

[B24] Slippers B. De Groot P. Wingfield M. J. (2012). *The Sirex Woodwasp and its Fungal Symbiont:: Research and Management of a Worldwide Invasive Pest.* Dordrecht: Springer Science & Business Media.

[B25] Song Y. Wang Y. Guo D. Jing L. (2019). Selection of reference genes for quantitative real-time PCR normalization in the plant pathogen *Puccinia helianthi* Schw. *BMC Plant Biol.* 19:20. 10.1186/s12870-019-1629-x 30634896 PMC6329156

[B26] Sun X. Tao J. Ren L. Shi J. Luo Y. (2016). Identification of *Sirex noctilio* (Hymenoptera: Siricidae) using a species-specific cytochrome C oxidase subunit I PCR assay. *J. Econ. Entomol.* 109 1424–1430. 10.1093/jee/tow060 27117170

[B27] Talbot P. (1977). The Sirex-amylostereum-pinus association. *Annu. Rev. Phytopathol.* 15 41–54.

[B28] Tang P. Duan J. Wu H. Ju X. Yuan M. (2017). Reference gene selection to determine differences in mitochondrial gene expressions in phosphine-susceptible and phosphine-resistant strains of *Cryptolestes ferrugineus*, using qRT-PCR. *Sci. Rep.* 7:7047. 10.1038/s41598-017-07430-2 28765619 PMC5539111

[B29] Tao Y. van Peer A. F. Huang Q. Shao Y. Zhang L. Xie B. (2016). Identification of novel and robust internal control genes from *Volvariella volvacea* that are suitable for RT-qPCR in filamentous fungi. *Sci. Rep.* 6:29236. 10.1038/srep29236 27405087 PMC4941408

[B30] Thompson B. M. Bodart J. McEwen C. Gruner D. S. (2014). Adaptations for symbiont-mediated external digestion in *Sirex noctilio* (Hymenoptera: Siricidae). *Ann. Entomol. Soc. Am.* 107 453–460.

[B31] Udvardi M. K. Czechowski T. Scheible W. R. (2008). Eleven golden rules of quantitative RT-PCR. *Plant Cell* 20 1736–1737. 10.1105/tpc.108.061143 18664613 PMC2518243

[B32] Vandesompele J. De Preter K. Pattyn F. Poppe B. Van Roy N. De Paepe A. (2002). Accurate normalization of real-time quantitative RT-PCR data by geometric averaging of multiple internal control genes. *Genome Biol.* 3 1–12. 10.1186/gb-2002-3-7-research0034 12184808 PMC126239

[B33] Wang G. Cheng H. Li M. Zhang C. Deng W. Li T. (2020). Selection and validation of reliable reference genes for *Tolypocladium guangdongense* gene expression analysis under differentially developmental stages and temperature stresses. *Genes* 734:144380. 10.1016/j.gene.2020.144380 31978511

[B34] Wang L. Li C. Shi J. Li C. Li J. Ren L. (2020). Incidental fungi in Host trees disrupt the development of *Sirex noctilio* (Hymenoptera: Siricidae) symbiotic fungus and larvae. *J. Econ. Entomol.* 113 832–838. 10.1093/jee/toz314 32253440

[B35] Wang L. Ren L. Li C. Gao C. Liu X. Wang M. (2019). Effects of endophytic fungi diversity in different coniferous species on the colonization of *Sirex noctilio* (Hymenoptera: Siricidae). *Sci. Rep.* 9:5077. 10.1038/s41598-019-41419-3 30911076 PMC6433867

[B36] Wang L. X. Ren L. L. Liu X. B. Shi J. Luo Y. (2018). Effects of endophytic fungi in Mongolian pine on the selection behavior of woodwasp (*Sirex noctilio*) and the growth of its fungal symbiont. *Pest Manag. Sci.* 75 492–505. 10.1002/ps.5146 30070049

[B37] Wang M. Wang L. Li D. Fu N. Li C. Luo Y. (2020). Advances in the study of mutualism relationship between *Amylostereum areolatum* and *Sirex noctilio*. *J. Temp. For. Res.* 3 1–11. 10.3969/j.issn.2096-4900

[B38] Wu Y. Zhang C. Yang H. Lyu L. Li W. Wu W. (2021). Selection and validation of candidate reference genes for gene expression analysis by RT-qPCR in *Rubus*. *Int. J. Mol. Sci.* 22:10533. 10.3390/ijms221910533 34638877 PMC8508773

[B39] Xiang Q. Li J. Qin P. He M. Yu X. Zhao K. (2018). Identification and evaluation of reference genes for qRT-PCR studies in *Lentinula edodes*. *PLoS One* 13:e0190226. 10.1371/journal.pone.0190226 29293626 PMC5749753

[B40] Xie F. Xiao P. Chen D. Xu L. Zhang B. (2012). miRDeepFinder: a miRNA analysis tool for deep sequencing of plant small RNAs. *Plant Mol. Biol.* 80 75–84. 10.1007/s11103-012-9885-2 22290409

[B41] Xu J. Xu Z. Zhu Y. Luo H. Qian J. Ji A. (2014). Identification and evaluation of reference genes for qRT-PCR normalization in *Ganoderma lucidum*. *Curr. Microbiol.* 68 120–126. 10.1007/s00284-013-0442-2 24013612

[B42] Yang M. Wu S. You W. Jaisi A. Xiao Y. (2019). Selection of reference genes for expression analysis in Chinese medicinal herb *Huperzia serrata*. *Front. Pharmacol.* 10:44. 10.3389/fphar.2019.00044 30774594 PMC6367274

[B43] Yang Z. Zhang R. Zhou Z. (2021). Identification and validation of reference genes for gene expression analysis in *Schima superba*. *Genes* 12:732. 10.3390/genes12050732 34068362 PMC8153319

[B44] Zhang X. Xu Z.-C. Xu J. Ji A.-J. Luo H.-M. Song J.-Y. (2016). Selection and validation of reference genes for normalization of quantitative real-time reverse transcription PCR analysis in *Poria cocos* (Schw.) Wolf (Fuling). *Chin. Med.* 11 1–17. 10.1186/s13020-016-0079-8 26937250 PMC4774131

[B45] Zhang Y. Yao F. Sun W. Fang M. Wu C. (2020). Screening of reference genes for qRT-PCR amplification in *Auricularia heimuer*. *Mycosystema* 39 1510–1519. 10.13346/j.mycosystema.200048

[B46] Zhao J. Zhou M. Meng Y. (2020). Identification and validation of reference genes for RT-qPCR analysis in Switchgrass under heavy metal stresses. *Genes* 11:502. 10.3390/genes11050502 32375288 PMC7291066

[B47] Zhao X. (2016). *Selection of Reference Genes for qRT-PCR in Wolfiporia cocos.* Ph.D. thesis, Wuhan: Wuhan Polytechnic University.

