# Supplementary material for: Identification and Validation of Reference Genes for Gene Expression Analysis in Different Development Stages of Amylostereum areolatum
Source: Front Microbiol. 2022 Jan 12;12:827241. doi: 10.3389/fmicb.2021.827241 (PMC8790227; doi:10.3389/fmicb.2021.827241)
Supplement: Supplementary file 1 [file Data_Sheet_1.docx]

**Supplementary Material**


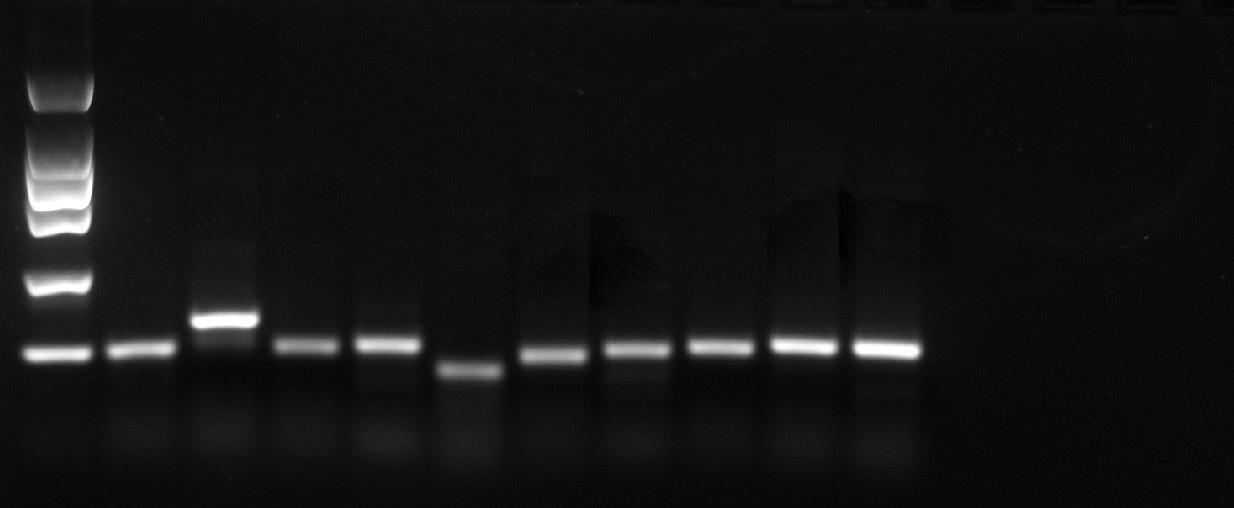


**bp**

**250**

***β-TUB***

**Marker**

***α-TUB***

***HH3***

***γ-TUB***

***CYP***

***GAPDH***

***MDP***

***CESP***

***CPR***

**100**

***P450***

**Figure S1** Amplified fragments of 10 candidate reference genes exhibited by agarose gel.


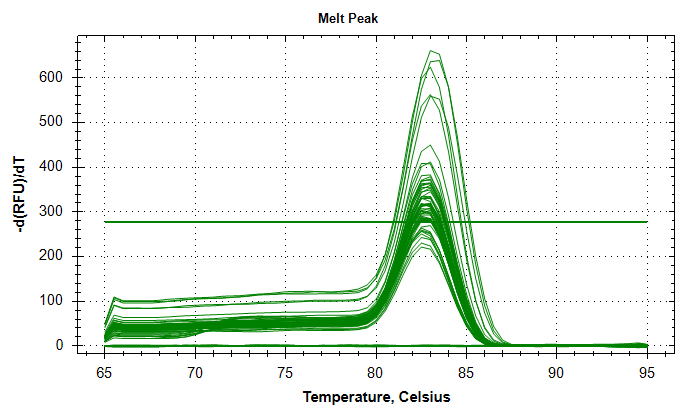


***β-TUB***


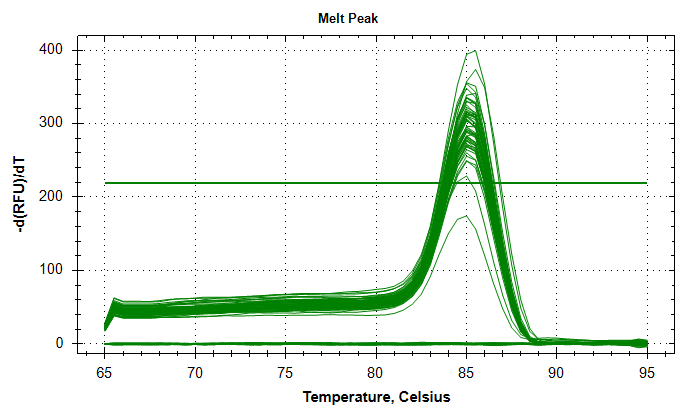


***α-TUB***


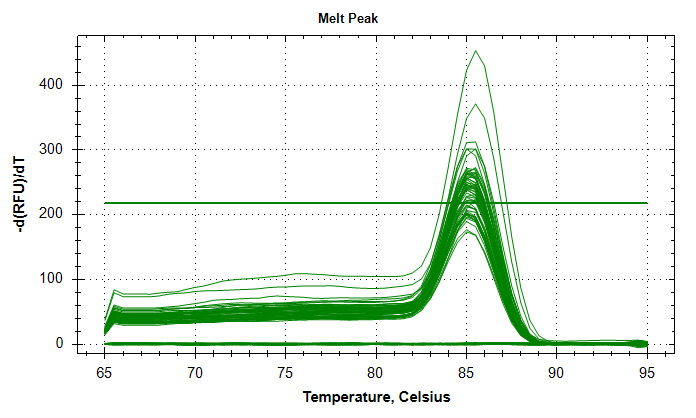


***HH3***


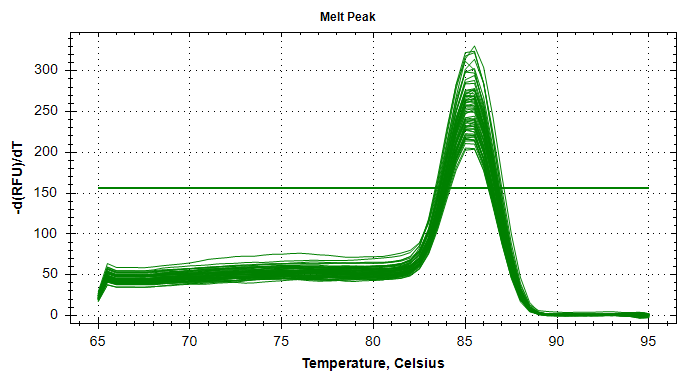


***γ-TUB***


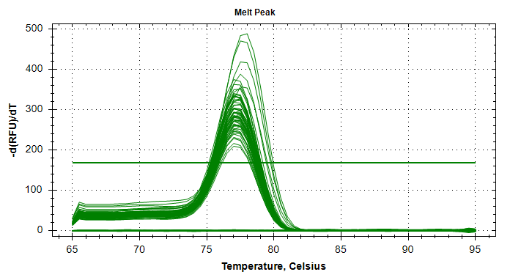


***CYP***


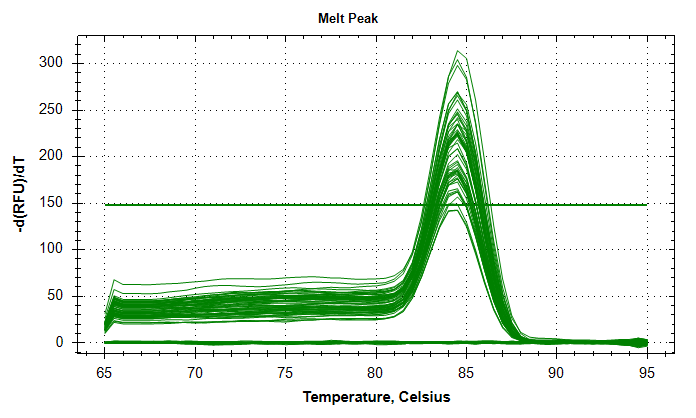


***GAPDH***


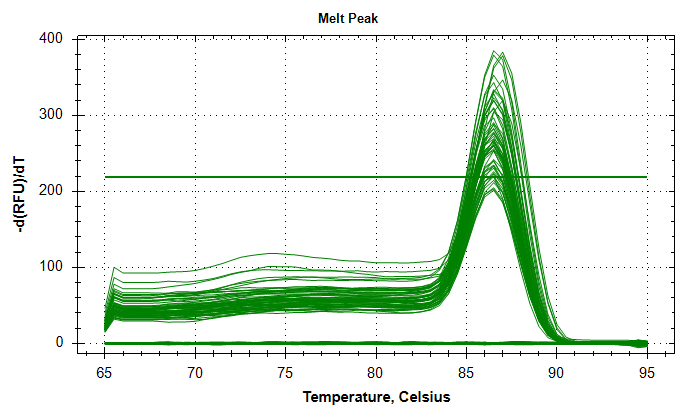


***MDP***


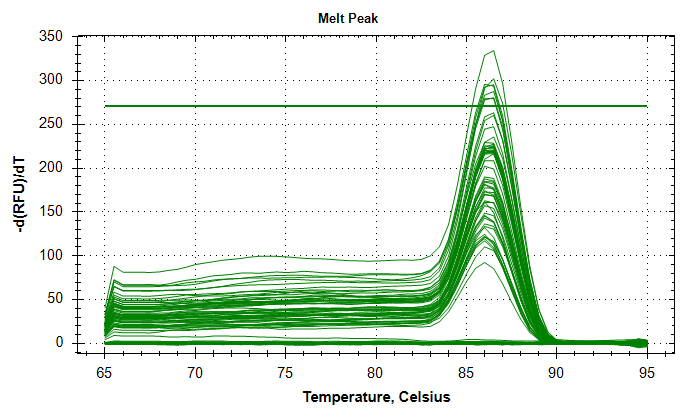


***CESP***


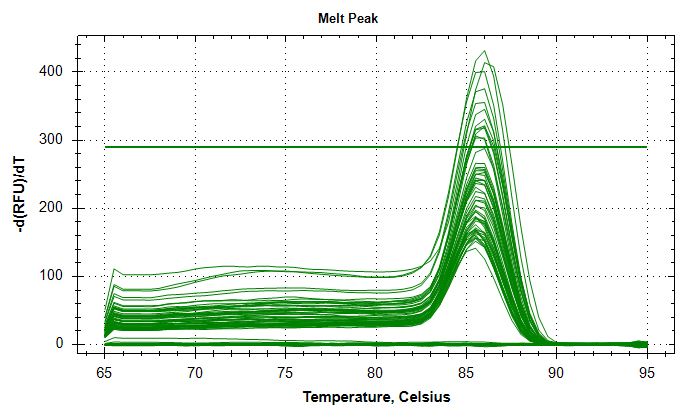


***CPR***


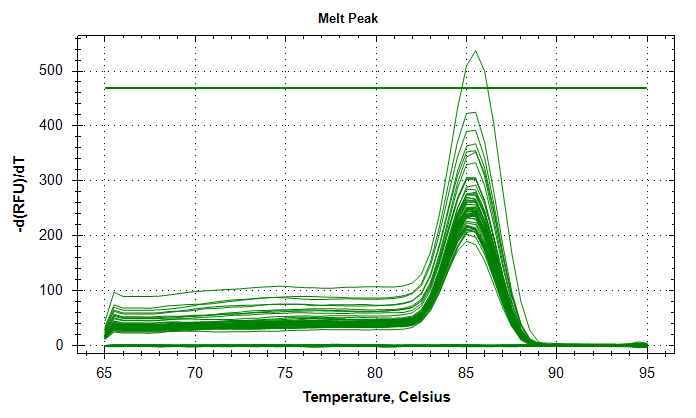


***P450***

**Figure S2** Melting curves for the candidate reference genes.
